# Risk Factors for Secondary Infertility among Women in Karachi, Pakistan

**DOI:** 10.1371/journal.pone.0035828

**Published:** 2012-04-27

**Authors:** Neelofar Sami, Tazeen Saeed Ali, Saba Wasim, Sarah Saleem

**Affiliations:** 1 Department of Community Health Sciences, Aga Khan University, Karachi, Pakistan; 2 School of Nursing, Aga Khan University, Karachi, Pakistan; University of Otago, New Zealand

## Abstract

**Background:**

Secondary infertility in developing countries is mostly attributable to blockage of the fallopian tubes due to adhesions caused by reproductive tract infections. There is a dearth of information on the prevalence and causes of secondary infertility from Pakistan. This paper presents results on factors associated with secondary infertility among married women in Karachi, Pakistan.

**Methods:**

A matched case-control study was conducted. Cases were women aged 15–35 years with history of at least one previous conception and currently seeking treatment for secondary infertility. Controls were women residing in the neighborhood of cases with at least one live birth and not taking treatment for secondary infertility. The age of controls was matched by ±5 years to that of cases. Data was collected from June to August 2003. Conditional logistic regression was used to determine crude and adjusted odds ratios (OR) with corresponding 95% confidence intervals (CI) for factors associated with secondary infertility.

**Results:**

The final multivariate logistic regression model revealed that after adjusting for age, cases were more likely to be the housewives (AOR = 2.6, 95% CI:1.5–4.4), had used inappropriate material to absorb blood during menstruation (AOR = 9.0, 95% CI: 5.0–16.4), and at their last delivery, had a birth attendant who did not wash hands with soap and water (AOR = 3.0, 95% CI: 1.4–5.7). Moreover, women with secondary infertility were more likely to report current or past history of having STI symptoms (AOR = 3.6, 95% CI: 2.4–5.6) and use of intra-vaginal indigenous medicines during their last post-partum period (AOR = 3.1, 95% CI: 1.6–5.7).

**Conclusion:**

We recommend health education and awareness messages for safe practices during menstruation, delivery, and the postpartum period for women in general. Additionally, sanitary napkins should be made available at an affordable cost, and safe delivery kits should contain educational/pictorial brochures for appropriate hand washing skills.

## Introduction

A couple is generally considered infertile if they are unable to achieve a clinical pregnancy after 12 months or more of regular unprotected sexual intercourse without use of contraceptives [1]. Primary infertility refers to couples who have never conceived whereas secondary infertility is usually defined as the inability to conceive for one year after having conceived at least once before [2]. Globally 10–15% of the couples are infertile and the secondary infertility out numbers the primary infertility [3]. The prevalence of primary and secondary infertility in Pakistan is nearly 5% and 18% respectively [4], [5].

The epidemiology of secondary infertility is not well understood [6], [7]. In developing countries, infections are the leading cause of secondary infertility among women [8]. Of these, sexually transmitted infections (STIs) [9], [10] as well as infections strewed through iatrogenic factors [11], [12], unsafe termination of pregnancy [13], [14], and unsafe birthing practices [15]–[17] have been identified as causes of secondary infertility in women from India, Bangladesh and Africa.

There is a dearth of information on prevalence of and factors associated with secondary infertility from Pakistan. However, there is evidence of unsafe practices commonly opted by service providers as well as by women during childbirth and postpartum period [16]–[18], resulting in pelvic inflammatory diseases (PID), tubal blockage and infertility. For example, more than 65 percent of the women are delivered in homes by unskilled birth attendants which expose them to infections [19], [20]. Other unsafe practices include lack of asepsis during insertion of IUCD, intra-vaginal placement of indigenous medicines and unsafe termination of pregnancy that could subsequently lead to infection and adhesions resulting in PID and secondary infertility [23], [24]. It has been proved that STIs, if not treated, can also result in secondary infertility and studies from Pakistan have reported STIs as not an uncommon entity [21], [22].

We present results on factors associated with secondary infertility in women in Karachi.

## Methods

A matched case control study was conducted in infertility clinics of five tertiary care hospitals from January to March 2003. A total of 17 hospitals providing services for management of infertility were approached. Of these, four public and one private hospital agreed to participate in the study. Women presenting to the outpatient departments of the infertility clinics were screened for the eligibility criteria. Women who had at least one previous conception irrespective of the outcome (i.e. a live birth, a still birth or a miscarriage) and were trying to conceive again for last one year were enrolled as cases. Controls were selected from the neighborhood of each case i.e. women living within five houses on either side of the household of the case with history of at least one live birth, stillbirth or a miscarriage but not taking any treatment to conceive again. The age of controls was matched by ±5 years to that of cases. Both cases and controls had the same spouse since their last pregnancy. As cases were selected from the facilities and were identified by the respective gynecologists, the response rate was 100%. Refusal rate among controls was 3 percent and a woman who refused was replaced by another woman in the next household. Prior to the interviews, cases and controls were asked for their informed written consent. The study was approved by the Ethical Review Board of Aga Khan University Hospital, Karachi Pakistan.

A structured questionnaire was developed and pretested before introducing to the study participants. All the interviews were conducted in local language “Urdu”. Cases were interviewed in the infertility clinics and controls were interviewed, within three days of the interview of cases, in their homes. Privacy was ensured while interviews were being conducted. The information was collected for demographics, use of contraceptives, practices during menstruation, previous deliveries and postpartum periods, and symptoms of reproductive tract infections.

For the study purpose, the following definitions were used:

Low risk place for delivery was defined as a hospital or a maternity home with delivery services while all other places such as homes and small clinics were considered as high risk place for delivery.

For conducting the delivery, doctors, midwives and Lady Health Visitors (LHVs) were considered as skilled birth attendants whereas Lady Health Workers (LHWs) and Traditional Birth Attendants (TBAs) were defined as unskilled attendants.

For absorbing lochia and/or menstrual blood, use of sanitary pads, new piece of cloth or washed piece of cloth dried in the sunlight was considered as appropriate practice. The use of cotton, unwashed rags and washed rags dried inside the room were considered as inappropriate practice.

Daily washing of perineum with water during peurperium was considered as an appropriate practice.

Insertion of home-made intra-vaginal preparations during peurperium was considered as an unsafe practice.

Women who reported present or past history of lower abdominal pain, vaginal discharge, urethral discharge, genital swelling, ulcers, blisters or any combination of these were considered as having symptoms of STIs. Women who reported their spouses having present or past history of urethral discharge, genital swelling, ulcers, blisters or any combination of these were considered as having symptoms of STIs.

For sample size estimation, we assumed the prevalence of the various risk factors for secondary infertility amongst the control group to be in the range of 10–60% [11], [18], [22], [25]. We took the option of taking a 1∶1 ratio between cases and controls to increase the power of the study. In order to be able to detect an odds ratio of at least 2 with a power of 80%, at a significance level of 5%, a sample size of 369 cases and 369 controls was calculated. Considering refusal rate of 8 percent the final estimated sample was at least 400 cases and 400 controls.

Conditional logistic regression was used to account for the matched case control study design. Analysis was performed using SAS version 9.2. Frequencies and percentages were computed for demographic and educational characteristics of cases and controls and their spouses. Univariate analysis was conducted for computing unadjusted matched ORs and their 95% confidence intervals. A multivariate parsimonious and biologically meaningful model was developed that best explained risk factors independently associated with secondary infertility in women of Karachi. Correlations between various variables were checked, and none of the interaction terms were found to be significant.

## Results

The mean age of cases and controls was similar (29.0±4.9 vs. 29.2±4.7 year). As compared to controls, a smaller proportion of women among cases was literate and employed (43.5% vs. 47.3% and 12.5% vs. 24.0% respectively). Similar trend was observed for literacy status of spouses of cases and controls. Compared to those of controls, husbands of infertile women were more illiterate (42.8% vs. 38.2%). However, the employment status of husbands was similar in both groups. (Table 1)

**Table 1:** Socio-Demographic characteristics of cases (n = 400) and matched controls (n = 400) studied for their association with secondary infertility in Karachi, Pakistan.

Variables	Cases	Controls
	n (%)	n(%)
**Respondent's Age (years)**	29.0±4.9	29.2±4.7
**Education respondent**		
0–5 years of schooling	227(56.8)	210(52.5)
6–10 years of schooling	131(33.0)	132(32.8)
11 years of schooling and above	42(10.5)	58(14.5)
**Education Husband**		
0–5 years of schooling	170(42.8)	153(38.2)
6–10 years of schooling	144(36.0)	165(41.3)
11 years of schooling and above	85(21.2)	82(20.8)
**Employment status of Respondent**		
House wife	350(87.5)	304(76.0)
Employed	50(12.5)	96(24.0)
**Employment Status of husband**		
Unemployed	20(5.0)	21(5.3)
Employed	380(95.0)	379(94.8)
**Duration of current marriage (years)**		
**<5**	42(10.5)	54(13.5)
**5–9**	169(42.2)	136(34.0)
**10–14**	112(28.0)	128(32.0)
**15 & above**	77(19.3)	82(20.5)

Univariate odds ratio (OR) with 95% confidence intervals (CI) for potential risk factors were evaluated for their association with secondary infertility (Table 2). On multivariate conditional logistic regression analysis (Table 3), women with secondary infertility were more likely to be the housewives (AOR = 2.6, 95% CI: 1.5–4.4). These women were more likely to have their last deliveries attended by unskilled birth attendants who did not wash their hands before conducting the deliveries (AOR = 3.0, 95% CI: 1.4–5.7) and had placed intra-vaginal indigenous medicines during the post-partum period. (AOR = 3.1, 95% CI: 1.6–5.7). Women with secondary infertility were more likely to have used inappropriate material to absorb blood during menstruation (AOR = 9.0, 95% CI: 5.0–16.4). They were almost 4 time more likely to have reported current or past history of symptoms of STIs (AOR = 3.6, 95% CI: 2.4–5.6) as compared to those of controls. Presence of symptoms of STIs among spouse lost significance on multivariate analysis.

**Table 2:** Univariate matched Odds Ratio (OR) and their 95% Confidence Intervals (CI) for risk factors evaluated for their possible association with secondary infertility in Pakistan (adjusted for age and area of residence).

Variables	Cases	Control	Crude Matched
	n = 400	n = 400	Odds Ratio(95% CI)
**Woman's Education**			
Illiterate and up to primary	227 (56.8)	210 (52.5)	1.2 (0.9–1.7)
Secondary	131 (32.8)	132 (33.0)	1.2(0.8–1.7)
Class 11 years and above	42(10.5)	58(14.5)	
**Husband's Education**			
Illiterate and up to primary	170(42.5)	152(38.0)	1.1(0.7–1.7)
Secondary	144(36.0)	165(41.3)	0.8(0.6–1.3)
Class 11 years and above	86(21.5)	83(20.8)	
**Woman's Employment status**			
House wife	350 (87.5)	304 (76.0)	2.4(1.6–3.7)
Employed	50(12.5)	96(24.0)	
**Husband's Employment status**			
Unemployed	20(5.0)	21(5.3)	0.9(0.5–2.0)
Employed	380(95.0)	379(94.8)	
**Birthing practices**			
High risk facility	218 (54.5)	152 (38.0)	2.1(1.5–2.8)
Unskilled birth attendant	131 (32.8)	95 (23.8)	1.6(1.2–2.3)
Not washing hands before delivery	164 (41.0)	69 (17.3)	4.3(3.0–6.4)
Clean sheets not used	128 (32.0)	35 (8.8)	5.0(3.2–7.9)
Clean instruments not used	186 (46.5)	150 (37.5)	1.5(1.1–2.0)
Gloves not used	156 (39.0)	87 (21.8)	2.6(1.8–3.6)
**Menstrual Practices**			
Inappropriate material used for absorbing blood	163 (40.8)	45 (11.3)	8.4(5.0–14.0)
Do not take bath	202(50.5)	177(44.3)	1.3(1.0–1.8)
**Postpartum Practices**			
Inappropriate material used for absorbing blood	84 (21.0)	60 (15.0)	1.7(1.1–2.5)
Use of intra-vaginal preparation	84 (21.0)	41 (10.3)	2.7(1.7–4.3)
**STIs**			
History of symptoms of STIs in respondents	251 (62.8)	166 (41.5)	2.8(2.0–4.0)
History of symptoms of STIs in spouse	62 (15.5)	34 (8.5)	2.1(1.3–3.3)

**Table 3:** Final multivariable conditional logistic regression model of risk factors associated with secondary infertility in Karachi, Pakistan.

Variables	Crude Matched Odds Ratio	Adjusted Matched Odds Ratio
	(95% CI)	(95% CI)
**Woman's Employment status**		
House wife	2.4(1.6–3.7)	2.6(1.5–4.4)
**Birthing practices**		
Not washing hands before delivery	4.3(3.0–6.4)	3.0(1.4–5.7)
Clean sheets not used	5.0(3.2–7.9)	2.0(0.9–3.9)
**Menstrual Practices**		
In appropriate material used for absorbing blood	8.4 (5.0–14.0)	9.0 (5.0–16.4)
**Postpartum Practices**		
Use of intra-vaginal preparation	2.7 (1.7–4.3)	3.1 (1.6–5.7)
**STIs**		
History of symptoms of STIs in respondents	2.8 (2.0–4.0)	3.6 (2.4–5.6)

## Discussion

The results of our study suggest that women with secondary infertility were more likely to be the house wives, and had carried out inappropriate practices for delivery, postpartum and menstrual care. Among the cases, 87% of women were housewives and 12.5% were employed as compared to 76% and 24% respectively for controls. Literacy status of women in both the groups was more or less similar and illiteracy was not significantly associated with secondary infertility.

Women's status at household level plays a central role for their health seeking behavior such as for making fertility choices and using contraceptives. [26]. In a patriarchal society such as Pakistan, generally men are considered as main bread winner while dominant roles for women are related to child bearing and taking care of household chores. The women's roles are irrespective of their literacy status. Literature from Pakistan suggests that choices for health care are limited for housewives and the situation gets graver if a woman is illiterate and unemployed too [27], [28].

In Pakistan more than 65% of the deliveries occur at homes and are attended by unskilled birth attendants. Most of these birth attendants are not aware of standard delivery protocol and opt for unsafe practices which could be harmful to both women and newborns [19]. One of the commonly recommended methods for safe delivery is to wash hands with soap and water before conducting delivery [29]–[31]. It is one of the simplest techniques to reduce chances of introducing infections but some technical training is needed to acquire the skills for washing hands. In our study, women with secondary infertility were four times more likely to have a birth attendant who did not wash her hands before conducting delivery. This needs behavior change not only on the part of service providers but for the clients too. Since most of the birth attendants conducting deliveries at homes are indigenous and illiterate, pictorial brochures with instructions should be made an integral part of delivery kits so that this practice can be adopted by every birth attendant. Additionally, the awareness sessions targeting the women could sensitize the clients and their relatives to keep a check for the appropriate hand washing practices of birth attendants.

Some practices during postpartum period are harmful for the women. Intra-vaginal placement of medicines is one of such traditions [18], [32], [33] and studies have shown that in South Asian regions, home-made vaginal preparations are commonly used during postpartum period for increasing the flow of ‘dirty blood’ from the body, bringing uterus in original position and flattening of abdomen [15], [17], [34]. These preparations are placed intra-vaginally by women themselves or by the unskilled birth attendants. Information on the health consequences of such practices is not well documented. Our study has identified use of homemade vaginal preparations as a risk factor for secondary infertility. The relation needs to be further explored for the role of indigenous substances per se, introduction of infection during the procedure or some other context.

Infections of reproductive tract are one of the main causes of infertility. Evidence for STIs as a cause of secondary infertility is available mostly from West Africa [35], [36]. The positive association of having symptoms of STIs and secondary infertility in our study should be interpreted with caution. We accepted women's responses at the face value and did not verify the disease. Studies have shown that physiological vaginal discharge is often considered pathological both by the respondent and the provider [37]. In our study ‘experiencing vaginal discharge’ was reported by a large number of women in both groups and hence the possibility of over reporting cannot be ruled out. However any bias caused by over reporting is distributed non-differentially among cases and controls and the calculated risk could be an underestimate.

Women of South Asia consider menstruation and postpartum period as unclean and grimy phases and generally refrain from adapting personal hygienic practices during these times. [38]. Culturally women abstain from taking bath during menstruation and 40 days after a childbirth [15], [39]. Additionally, the use of inappropriate material for absorbing blood and lochia during menstruation and postpartum period respectively is not uncommon. The rags are reused after washing and drying. The latter is done secretly in the rooms away from sunlight as menstruation is thought to be a shameful act. All such unhygienic practices could be a source of infection ascending through vagina to uterus and fallopian tubes causing adhesions ending up in secondary infertility. However more research is needed to prove this hypothesis.

Our criteria for selection of controls were based on having at least one live birth, still birth or a miscarriage but not taking treatment for secondary infertility. There is a possibility that some of these women were having secondary infertility not known to them at the time of enrollment into the study as controls. We did not perform any investigations to confirm their control status. Such bias in selection of controls would have resulted in underestimation of the risks.

In our study the interval between women's last childbirth and seeking treatment for secondary infertility was 3.6±1.8 years. Hence there is a probability of recall bias on the part of the woman to remember the practices carried out by the birth attendant at the time of last delivery. We believe that this information provided by the women is non-differentially distributed among cases and control and the estimated risks are an underestimate.

In conclusion the modifiable risk factors associated with secondary infertility were unsafe practices during delivery, postpartum period, and menstruation. Presence of symptoms of sexually transmitted infections in women was also associated with secondary infertility. We recommend that women in general should have appropriate health education and awareness about safe practices during delivery. All delivery kits should contain educational and pictorial brochures for appropriate hand washing skills. Such posters should also be placed at health facilities too. Raising awareness regarding hygienic practices during menstruation and postpartum period has remained largely a neglected area in developing countries. In addition to highlighting the importance of cleanliness during these time periods, availability of cheap safe sanitary napkins and messages for their use should be given due importance and be included in public health messages.
